# Editorial: Immunoregulation and inflammation interventions in nutritional metabolic diseases

**DOI:** 10.3389/fnut.2026.1932965

**Published:** 2026-07-23

**Authors:** Lixue Wang, Lingli Zhou, Dachuan Dong, Yuhuai Xie

**Affiliations:** 1 Department of Basic Veterinary Medicine, College of Veterinary Medicine, Shandong Agricultural University, Tai'an, China; 2 Hunan Vocational College of Science and Technology, Changsha, China; 3 Division of Nephrology, Department of Medicine, Stanford University, Palo Alto, CA, United States; 4 Geriatric Research Education and Clinical Center, Veterans Administration Palo Alto Health Care System, Palo Alto, CA, United States; 5 Department of Immunology, School of Basic Medical Sciences, Fudan University, Shanghai, China

**Keywords:** chronic inflammation, gut microbiota, immunometabolism, nutritional indices, nutritional metabolic diseases

Nutritional metabolic diseases, including obesity, type 2 diabetes, non-alcoholic fatty liver disease (NAFLD), metabolic dysfunction-associated steatotic liver disease (MASLD), and cardiovascular diseases, are increasingly recognized as chronic, low-grade inflammatory conditions linked to metabolic dysregulation (1, 2). Emerging evidence suggests that nutritional status may modulate inflammatory responses, restore immune homeostasis, and thereby improve metabolic outcomes (3, 4). This shifted research focus highlighted the intricate interplay between systemic inflammation and nutritional status. This Research Topic brings together 13 articles that cover a wide range of studies exploring novel diagnostic tools, molecular drivers, and bioactive interventions, collectively advancing our understanding of how immunoregulation and anti-inflammatory interventions can improve metabolic outcomes, while also identifying critical knowledge gaps for future investigations. Several themes emerged across these diverse contributions.

A central focus of several studies in this Research Topic is the interplay between gut microbiota and host inflammation. Men et al. demonstrated that a novel functional water, devoid of any additives, significantly increased gut microbial diversity and enriched beneficial genera such as *Akkermansia* and *Faecalibaculum* while reducing pro-inflammatory cytokines IL-1β, IL-6, and TNF-α in healthy mice. This study introduces an affordable, safe, non-pharmacological strategy for basal inflammation reduction.

Extending this microbial modulation concept to established metabolic diseases, Huang et al. showed that hyperoside, a plant-derived flavonoid, ameliorated NAFLD in rats by remodeling gut microbiota (enriching *Lactobacillus*, suppressing *Escherichia-Shigella*) and reprogramming serum metabolic networks, with beneficial bacteria negatively correlating with pro-inflammatory metabolites. Complementing this, Dong et al. employed an integrated microbiota-metabolomics approach to demonstrate that ursodeoxycholic acid (UDCA) alleviates high-fat diet-induced liver injury through gut microbiota-mediated bile acid metabolism, activating PPARγ/Nrf2 antioxidative signaling while inhibiting NF-κB-mediated inflammation. Collectively, these three studies underscore that targeting the gut microbiota–inflammation axis, whether through functional water, phytochemicals, or bile acid-based therapeutics, holds promise for managing nutritional metabolic diseases.

Nutritional indices serve not only as predictors of metabolic disease risk but also as modifiable therapeutic targets. Cheng et al. conducted a large-scale cross-sectional study of 10,901 Chinese adults and demonstrated that three simple nutritional indices, namely Geriatric Nutritional Risk Index (GNRI), Controlling Nutritional Status (CONUT), and Prognostic Nutritional Index (PNI), effectively predict the risk of MASLD. Notably, GNRI showed superior predictive performance (AUC = 0.909), particularly in women, young individuals (under 35 years), and those with normal body mass index. This finding offers a low-cost screening tool applicable in resource-limited settings.

The clinical relevance of nutritional status extends to liver disease prognosis. Li et al. in a systematic review and meta-analysis of 11 cohort studies with 2,072 participants, demonstrated that sarcopenia is significantly associated with an increased mortality risk in acute-on-chronic liver failure (ACLF) patients (HR = 1.27). This association was even stronger for short-term mortality (HR = 1.54), reinforcing that muscle mass, as a modifiable nutritional outcome, directly impacts survival in advanced liver disease.

Addressing the translational gap between mechanistic evidence and clinical application, a particularly valuable contribution to this topic comes from Russo et al., who conducted a systematic review on vitamin D and resveratrol in sarcopenic obesity (SO). Despite compelling mechanistic evidence linking these compounds to NF-κB signaling, PGC-1α, and mitochondrial regulation, their review identified zero phenotype-defined randomized controlled trials that required baseline confirmation of both adiposity and sarcopenia as enrollment criteria. This finding serves as an important cautionary note: mechanistic plausibility does not guarantee clinical evidence, and future RCTs must adopt rigorous phenotype-defined enrollment strategies.

The translational potential of immunometabolism research is exemplified by a study that bridges basic findings to clinical practice. Zhang et al. developed and prospectively validated a dynamic nomogram integrating immuno-nutritional markers (systemic inflammation response index, SIRI; Nutritional Risk Screening 2002, NRS-2002) with glycemic control (HbA1c) to predict abdominal compartment syndrome in diabetic patients undergoing complex ventral hernia repair. The model demonstrated robust discrimination in both the training cohort (AUC = 0.89) and prospective temporal validation (AUC = 0.84), illustrating how composite indices of inflammation and nutrition can be translated into actionable bedside tools.

Collectively, articles in this Research Topic have advanced our understanding of immunoregulation and inflammation interventions in nutritional metabolic diseases from multiple perspectives. They have provided novel insights into gut microbiota as a therapeutic target, validated simple nutritional indices for disease risk stratification, critically identified the lack of phenotype-defined trials, and demonstrated the translational potential of immuno-nutritional markers in clinical prediction. These findings collectively underscore the value of integrating immunological and nutritional perspectives in both mechanistic research and clinical practice. Whether these mechanistic insights can be translated into precision nutrition strategies tailored to individual immune and metabolic profiles remains an open question.

Despite these advancements, several challenges persist. The optimal strategies for gut microbiota modulation and their long-term effects remain to be defined. The striking absence of phenotype-defined RCTs, as highlighted by Russo et al., likely extends to other nutritional metabolic disease phenotypes and calls for urgent attention. Addressing these gaps through well-designed studies will be essential to translate current mechanistic insights into actionable interventions for patients.
